# AAB-FusionNet: A real-time object detection model for UAV edge computing platforms

**DOI:** 10.1016/j.mex.2025.103654

**Published:** 2025-09-26

**Authors:** Chi Kien Ha, Hoanh Nguyen, Long Ho Le

**Affiliations:** Faculty of Electrical Engineering Technology, Industrial University of Ho Chi Minh City, Ho Chi Minh City 700000, Vietnam

**Keywords:** Object detection, Edge computing platforms, Unmanned aerial vehicles, Feature fusion

## Abstract

Unmanned aerial vehicles (UAVs) often operate under stringent resource constraints while requiring real-time object detection, which can lead to failures in cluttered backgrounds or when targets are small or partially occluded. To address these challenges, we introduce AAB-FusionNet, a real-time detection model specifically designed for UAV edge computing platforms. At its core is the Adaptive Attention Block (AAB), which employs an Adaptive Saliency-based Attention (ASA) mechanism to highlight the most discriminative tokens while a lightweight MBConv sub-layer refines local spatial features. This saliency-driven framework ensures the network remains focused on critical cues despite complex aerial imagery. To further boost performance, AAB-FusionNet utilizes a Multi-layer Feature Fusion Network that integrates three key components: Attentive Inverted Bottleneck Aggregation (AIBA) to restore significant details at multiple scales, DySample for preserving spatial fidelity during feature alignment, and the Dual-Attention Noise Mitigation (DNM) module to suppress environmental noise through complementary channel and spatial attention. Experiments on diverse aerial datasets confirm that AAB-FusionNet achieves robust detection, especially for small or partially occluded objects, while offering real-time inference on low-power hardware. Overall, AAB-FusionNet effectively balances accuracy, computational efficiency, and adaptability, making it ideally suited for UAV scenarios demanding fast, reliable object detection and robust and consistent performance.

• Incorporates an Adaptive Saliency-based Attention mechanism to emphasize critical visual cues.
• Introduces a Multi-layer Feature Fusion Network for detail restoration, feature alignment, and noise mitigation.
• Demonstrates real-time, high-accuracy detection on low-power UAV platforms, particularly for small or occluded targets.

## Graphical abstract

**Figure:**
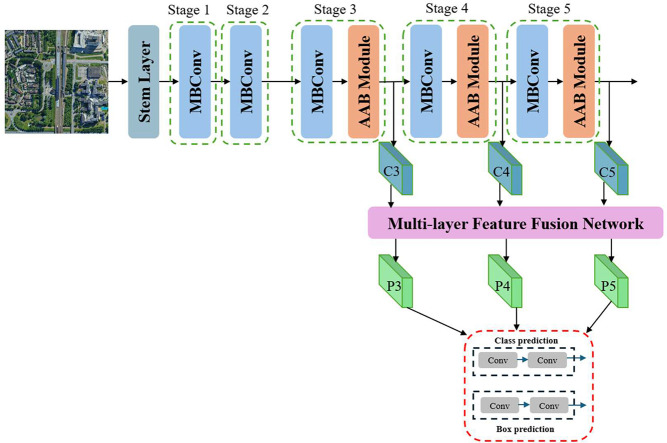

## Specifications table

**Table:** 

**Subject area**	Computer Science
**More specific subject area**	*Computer Vision, Deep Learning*
**Name of your method**	*AAB-FusionNet*
**Name and reference of original method**	*None*
**Resource availability**	*We evaluate our proposed model using two publicly available remote sensing datasets: DIOR and RSOD.*

## Background

Unmanned Aerial Vehicles (UAVs) have become indispensable in numerous real-world applications, including aerial surveillance, search and rescue, precision agriculture, and traffic monitoring [1,2]. Their agility and ability to operate in diverse, often remote environments enable them to gather critical data and carry out missions that would be arduous or dangerous for human operators. In these contexts, precise object detection facilitates timely decision-making and fosters safer, more efficient task execution. By providing visual insights in near real time, UAV-based object detection systems expand operational possibilities in a variety of industries, from environmental science to public safety. Real-time object detection plays a pivotal role in enabling UAVs to rapidly perceive and respond to dynamic environments. Whether tracking moving objects, avoiding obstacles, or assessing changing conditions, the capacity to identify critical elements in the scene within tight latency constraints ensures that UAVs can execute complex maneuvers autonomously. Furthermore, instant feedback is essential for applications like disaster response, where delays could compromise rescue operations or endanger human lives. As UAVs increasingly replace or augment traditional ground-based systems, the demand for high-performing, low-latency detection methods continues to grow.

Deploying standard detection frameworks on resource-limited UAV edge devices introduces significant challenges. Onboard processors often have limited memory, computational speed, and power availability, making it difficult to run large-scale neural networks without noticeable performance degradation. These constraints can lead to trade-offs where maintaining desired frame rates comes at the cost of reduced accuracy or truncated input resolutions. Overcoming such limitations demands targeted innovations in network design that can adapt to constrained hardware while preserving reliable detection capabilities. State-of-the-art detection networks, while accurate, typically involve high computational and memory demands that exceed the capabilities of onboard processors. Approaches such as Faster R-CNN [3], SSD [4], and more recent transformer-based models often excel on benchmark datasets when deployed on powerful GPUs but underperform dramatically on embedded devices. This discrepancy highlights the need for architectures specifically designed with hardware efficiency in mind. Additionally, elaborate feature extractors and large parameter counts exacerbate latency issues, ultimately restricting the operational envelope of UAV-based detection systems in scenarios where real-time performance is critical.

Moreover, aerial imagery often contains small, partially occluded objects against cluttered backgrounds, further complicating detection tasks and exacerbating performance bottlenecks. UAV cameras typically have wide fields of view, resulting in diminished pixel coverage for distant targets. Rapid viewpoint changes and complex terrain features may also introduce occlusions, making it difficult to identify objects based on partial or noise-corrupted cues. These intricate conditions emphasize the importance of multi-scale analysis and robust feature extraction, especially if detection remains subject to real-time constraints. Existing lightweight models, such as MobileNet-based or ShuffleNet-based detectors, offer some relief by reducing complexity, but may struggle to capture both the global context and fine-grained details required for reliable detection of small targets in complex UAV scenes. Their streamlined architectures often sacrifice some representational power, potentially leading to false negatives when objects appear in challenging poses or at small scales. Despite these improvements, the fundamental difficulty lies in combining efficiency with the capacity to handle the large spatial variations and occlusions typical in aerial videos. Additionally, noise from environmental factors like wind, glare, and atmospheric distortions can degrade feature representations, underscoring the need for a robust approach that effectively filters irrelevant information while emphasizing critical cues in real time.

Convolutional neural networks (CNNs) have long been recognized as the workhorse of object detection, following the success of Faster R-CNN and SSD. These frameworks demonstrated impressive detection accuracy but often incurred high computational costs, posing hurdles for deployment on UAV edge devices. To mitigate these efficiency concerns, single-stage models like the YOLO (You Only Look Once) series [[5], [6], [7], [8], [9], [10], [11], [12], [13]] progressively struck better trade-offs between inference speed and detection quality. Techniques such as anchor-free prediction [14], feature pyramid networks [15], and improved loss functions further enhanced performance while retaining real-time capabilities. Lightweight backbones such as MobileNet [16], ShuffleNet [17], SqueezeNet [18], and EfficientNet [19] leveraged depthwise separable convolutions, inverted bottlenecks, and compound scaling to reduce complexity. These efforts facilitated deployment on resource-constrained platforms, though challenges remain in capturing long-range dependencies and handling cluttered UAV scenes.

More recently, transformer-based models have emerged as an alternative due to their ability to capture global relationships within images. DETR (Detection Transformer) [20] pioneered this paradigm by formulating detection as a direct set prediction problem using multi-head self-attention and bipartite matching. Although eliminating hand-crafted anchors, DETR initially suffered from slow convergence and weak performance on small objects. Deformable DETR [21] introduced multi-scale deformable attention to mitigate these issues, while Sparse DETR [22] and Conditional DETR [23] further improved efficiency. Hybrid CNN-transformer approaches [24] and lightweight transformer modules [25] attempt to combine global context modeling with computational efficiency. Nevertheless, direct deployment of transformer-based frameworks on UAV edge devices remains challenging due to their high complexity.

In response to these limitations, we propose AAB-FusionNet, a real-time object detection model for UAV edge computing platforms. Our framework integrates attention-driven saliency modules within a lightweight CNN backbone and multi-stage feature fusion. Specifically, we introduce the Adaptive Attention Block (AAB), combining Adaptive Saliency-based Attention (ASA) and MBConv sub-layers to balance global context and fine-grained details. Furthermore, we design a Multi-layer Feature Fusion Network, leveraging Attentive Inverted Bottleneck Aggregation (AIBA), DySample, and Dual-Attention Noise Mitigation (DNM) modules to enhance multi-scale feature representation while mitigating noise. Together, these innovations provide a practical balance between accuracy and efficiency under UAV hardware constraints. Our contributions can be summarized as:

- We design AAB-FusionNet, a real-time object detection model for UAV edge computing platforms, incorporating specialized attention mechanisms, multi-scale feature fusion, and noise mitigation strategies to tackle demanding aerial perception tasks.
- We introduce an Adaptive Attention Block (AAB), which combines Adaptive Saliency-based Attention (ASA) and MBConv sub-layers, allowing the detector to focus on salient regions and boost representational capacity without incurring substantial computational overhead.
- We implement a Multi-layer Feature Fusion Network that leverages Attentive Inverted Bottleneck Aggregation (AIBA), DySample, and Dual-Attention Noise Mitigation (DNM) modules to manage multi-scale representations, preserve spatial alignment, and reduce noise artifacts in aerial imagery.
- Comprehensive experiments and ablation studies demonstrate that AAB-FusionNet achieves superior accuracy-latency trade-offs on UAV-specific benchmarks, outperforming several state-of-the-art detectors under resource-constrained settings.

## Method details

### Hardware and software environment

To ensure reproducibility and practical deployment feasibility, we provide a detailed description of the computational infrastructure and software environment used for both training and testing AAB-FusionNet. The training stage was performed on a high-performance workstation equipped with a modern GPU, multi-core CPU, and ample system memory, while deployment was validated on an embedded platform tailored for UAV edge computing. This dual-level evaluation guarantees that the proposed framework is not only feasible under high-resource research settings but also operationally viable in real-world UAV scenarios, where efficiency and power constraints are critical. The primary server environment featured an NVIDIA RTX 4080 GPU (16 GB VRAM) to accommodate high-resolution datasets, paired with an AMD Ryzen 9 5950X CPU (16 cores) and 128 GB of DDR4 RAM to ensure smooth preprocessing and parallel data handling. For embedded validation, we selected the NVIDIA Jetson Orin Nano, which integrates 32 Tensor Cores with FP16 support, offering a balance between low power consumption and sufficient throughput for real-time inference. Both environments ran on Ubuntu 22.04 LTS, utilizing PyTorch 2.1, CUDA 12.2, and cuDNN 8.9 to ensure compatibility with widely adopted deep learning toolchains. Optimization was conducted using the AdamW optimizer with a cosine learning rate scheduler, chosen for its proven stability in training both CNN and transformer-based detectors. Table 1 summarizes the computational environment, clearly delineating the resources required for both development and deployment phases.

**Table 1:** Computational environment.

Component	Specification
GPU (server)	NVIDIA RTX 4080 (16 GB VRAM)
CPU	AMD Ryzen 9 5950X (16 cores)
RAM	128 GB DDR4
Embedded device	NVIDIA Jetson Orin Nano (32 Tensor Cores, FP16 support)
OS	Ubuntu 22.04 LTS
Framework	PyTorch 2.1, CUDA 12.2, cuDNN 8.9
Optimization	AdamW optimizer, Cosine LR scheduler

### Data preparation

We evaluated AAB-FusionNet using two publicly available remote sensing datasets: DIOR [29] and RSOD [30].

#### DIOR

Proposed by Han Jw’s team at XIT, DIOR comprises 23,463 images (800 × 800 pixels) with 192,472 annotations across 20 object categories: airplane (AL), airport (AT), baseball field (BF), basketball court (BC), bridge (B), chimney (C), dam (D), expressway service area (ESA), expressway toll station (ETS), harbor (HB), golf course (GC), ground track field (GTF), overpass (O), ship (S), stadium (SD), storage tank (ST), tennis court (TC), train station (TS), vehicle (V), and windmill (WM). The dataset is partitioned into training (5862 images), validation (5863), and testing (11,725), providing a structured benchmark for generalization across diverse aerial targets.

#### RSOD

RSOD contains 2326 high-resolution images (1044 × 915 to 1288 × 992 pixels) with four categories: oil tanks, aircraft, overpasses, and playgrounds. A 5:1 train/validation split is used. To augment data diversity, images were transformed through translation, scaling, and rotations (90°, 180°, 270°). These augmentations are particularly beneficial for classes with rotational symmetry, such as oil tanks.

#### Preprocessing and standardization

All images were normalized using ImageNet mean and standard deviation. Annotations were converted into COCO-style JSON format for compatibility with PyTorch training pipelines. Both datasets underwent random cropping, horizontal/vertical flips, and color jitter during training to improve model robustness. This preparation pipeline ensures reproducibility and enables direct reuse for other aerial detection tasks.

### Framework overview

**Fig. 1:**
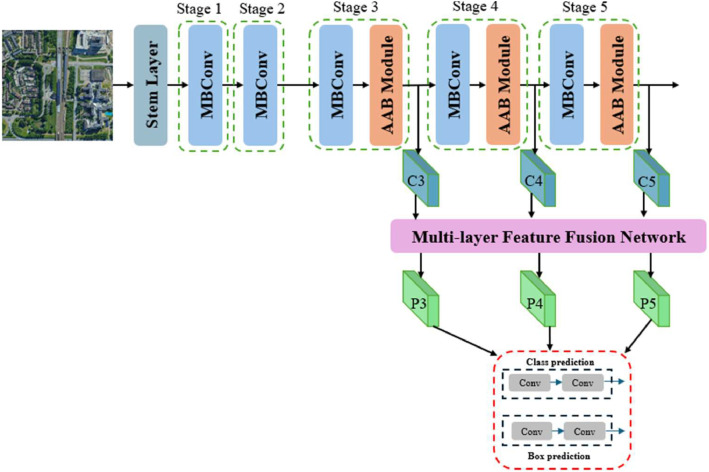
Schematic of AAB-FusionNet, illustrating the backbone, multi-scale feature fusion modules, and final detection heads tailored for UAV edge computing.

Fig. 1 provides overall architecture of the AAB-FusionNet. Our real‐time UAV object detection framework processes aerial images in a streamlined, end‐to‐end manner that balances accuracy with the strict computational constraints of edge devices. First, a stem layer ingests the raw input and produces low‐level feature maps with minimal spatial reduction. These features then traverse five sequential stages. Stages 1 and 2 consist solely of MBConv blocks [26], which apply depthwise‐separable convolutions and channel expansion to learn basic spatial‐channel representations. Starting from Stage 3, each block is augmented with an Adaptive Attention Block (AAB), combining Adaptive Saliency‐based Attention (ASA) and an MBConv submodule. The ASA mechanism identifies and aggregates the most discriminative tokens, assigning higher saliency weights to key features. This saliency‐aware global‐context modeling helps the network focus on pivotal regions while filtering out irrelevant background clutter. Subsequently, the MBConv submodule refines local‐spatial details, ensuring that each stage outputs features with both global and local context.

The refined feature maps from different stages are fed into the Multi‐layer Feature Fusion Network, where three specialized modules orchestrate fusion and enhancement. Specifically, each feature scale first undergoes the Attentive Inverted Bottleneck Aggregation (AIBA) module for spatial‐channel refinement, assisted by partial convolutions and efficient channel attention. Next, a DySample operation aligns the features across resolutions for effective top‐down or bottom‐up fusion, followed by the Dual‐Attention Noise Mitigation (DNM) module, which applies parallel dilated depthwise convolutions and channel attention to suppress environmental noise. Finally, the resulting fused feature maps are passed to the detection heads, comprising class and box prediction branches, for final object detection outputs. By integrating ASA within the backbone and leveraging multi‐scale feature enhancement through AIBA, DySample, and DNM, our model handles the complexities of UAV aerial data under real‐time constraints. This comprehensive design ensures that salient cues are highlighted early on, noise is mitigated, and multi‐scale information is systematically fused before the final detection stage.

### Adaptive attention block

**Fig. 2:**
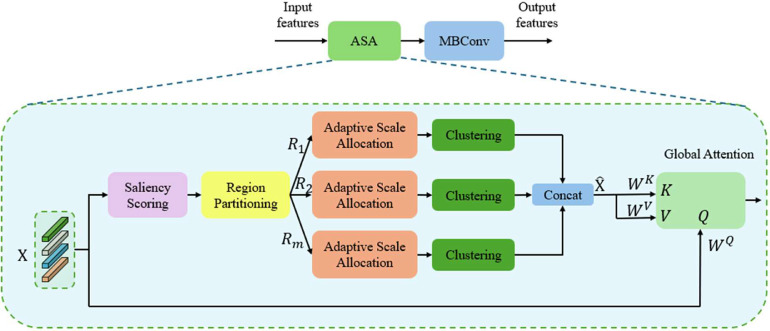
Detailed layout of the Adaptive Attention Block (AAB), showing how the Adaptive Saliency-based Attention (ASA) mechanism interfaces with MBConv layers to emphasize critical tokens.

Real‐time object detection in aerial imagery often faces challenges of cluttered backgrounds and rapid viewpoint changes. A mechanism that adaptively focuses attention on salient regions while merging redundant features is therefore crucial for efficiency. Based on this observation, the Adaptive Attention Block (AAB) is designed to balance robust context modeling with minimal computational overhead on UAV edge devices. Fig. 2 shows the detailed structure of the AAB. It consists of two main components: the Adaptive Saliency‐based Attention (ASA) module and an MBConv sub‐layer. Input features first enter ASA, which identifies and aggregates salient tokens through a combination of region partitioning, adaptive scale allocation, and clustering. The aggregated tokens then undergo a global attention operation that fuses salient features with the original inputs. The output of ASA is passed on to MBConv for further local‐spatial refinement. This combination of ASA and MBConv elevates the representational capacity of the network while preserving real‐time performance.

In the ASA module, the model adaptively identifies the most discriminative tokens (e.g., regions that help the detector focus on critical cues in real‐time UAV imagery). By assigning higher saliency to key tokens, the network avoids devoting excessive attention to uninformative background elements.

Let $X\in R^{N\times d}$ denote the set of N input tokens, each of dimensionality d. A saliency scoring network produces:

(1) $$s={\left[s_{1},s_{2},\ldots ,s_{N}\right]}^{T},s_{i}=\emptyset \left(x_{i}\right)$$

where $x_{i}\in R^{d}$ is the ith token, and ∅(.) is a lightweight multi‐layer perceptron (MLP). The scalar $s_{i}$ captures the importance or saliency of each token for subsequent feature processing.

After scoring, tokens are sorted by their saliency values and subdivided into M irregular sub‐regions ${\left\{R_{m}\right\}}_{m=1}^{M}$. Each sub‐region $R_{m}$ contains tokens with comparable saliency ranges. An aggregation scale $\gamma_{m}$ is then assigned to each $R_{m}$ using normalized density factors:

(2) $$\rho_{m}=\psi \left(R_{m}\right)$$

(3) $${\hat{\rho }}_{m}=\frac{e^{\rho_{m}}}{\sum_{k=1}^{M}e^{\rho_{k}}}$$

(4) $$c_{m}=N_{A}{\hat{\rho }}_{m}$$

(5) $$\gamma_{m}=\frac{N_{R_{m}}}{c_{m}}$$

where ψ(.) is a small MLP that outputs $\rho_{m}$, $c_{m}$ is the number of aggregated tokens in region m, and $N_{A}$ is a predefined budget for aggregated tokens. Sub‐regions with strong saliency (e.g., an object boundary) receive smaller aggregation scales $\gamma_{m}$, preserving more tokens for fine details; less critical areas are merged more aggressively.

Within each sub‐region $R_{m}$, we cluster the tokens into $c_{m}$ groups via a density‐peak clustering algorithm [27]. Each cluster $C_{m,k}$ yields a cluster‐level representation:

(6) $$y_{m,k}=\frac{\sum_{x_{j}\in C_{m,k}}e^{\eta s_{j}}x_{j}}{\sum_{x_{j}\in C_{m,k}}e^{\eta s_{j}}}$$

where η is a learnable temperature controlling the influence of each token’s saliency $s_{j}$. All resulting cluster centroids $y_{m,k}$ are concatenated to form a reduced set of aggregated tokens $\hat{X}\in R^{N_{A}\times d}$ .

Clustering helps eliminate redundant features often present in UAV imagery while preserving each cluster’s essential representation. Saliency weighting guarantees that more discriminative features have a stronger effect on the final cluster embeddings.

Finally, global attention operates between the original tokens X and these aggregated tokens $\hat{X}$. We define:

(7) $$Q=XW^{Q},\;V=\hat{X}W^{V},\;K=\hat{X}W^{K}$$

where $W^{Q},W^{V},\;W^{K}\in R^{d\times d}$ are learnable parameters. Then the final output is:

(8) $$ASA\left(X\right)=Softmax\left(\frac{QK^{T}}{\sqrt{d}}\right)V$$

Here, Q acts as queries, while K and V are keys and values derived from the aggregated token set $\hat{X}$. The final attention output is passed to the MBConv sub‐module, capturing local‐spatial information and refining features further. By combining ASA for global‐context modeling with MBConv for local refinement, the AAB provides an efficient, saliency‐aware feature extractor well suited for real‐time UAV object detection.

### Multi-layer feature fusion network

**Fig. 3:**
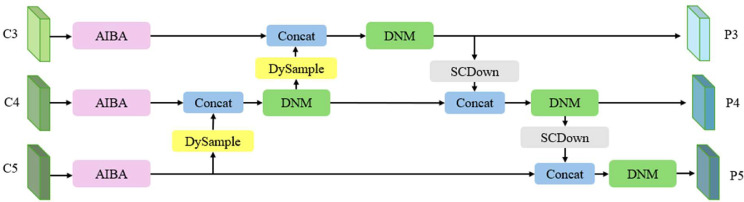
Multi-layer Feature Fusion Network, featuring Attentive Inverted Bottleneck Aggregation (AIBA), DySample for resolution alignment, and the Dual-Attention Noise Mitigation (DNM) module for noise suppression.

In our multi‐layer feature fusion design (Fig. 3), each backbone output C3, C4, and C5 first enters the Attentive Inverted Bottleneck Aggregation (AIBA) module, which employs an inverted bottleneck with partial convolutions and efficient channel attention to refine and restore crucial details in the feature maps. Next, a DySample operation [28] adaptively resizes and aligns these features across scales, preserving spatial integrity for subsequent fusion. Once properly aligned, features from multiple scales are concatenated and passed into the Dual‐Attention Noise Mitigation (DNM) module. The DNM simultaneously applies channel attention and spatial attention, via parallel depthwise convolutions with different dilation rates, to suppress environmental noise and highlight target‐relevant regions. Finally, if further spatial downsampling is required, an SCDown [13] block reduces the resolution of the fused feature maps, ensuring that multi‐scale information is efficiently propagated through the network. By orchestrating these modules, the multi‐layer feature fusion network continuously produces high‐quality, noise‐reduced feature maps across scales, ultimately improving detection accuracy under real‐time UAV operating constraints.

### Attentive inverted bottleneck aggregation module

To strengthen the multi‐scale feature fusion in our neck and improve robustness under challenging UAV imaging conditions, we introduce an Attentive Inverted Bottleneck Aggregation (AIBA) module. Placed at the interface between the backbone outputs and the subsequent neck layers, the AIBA module consolidates feature maps from different backbone stages and refines them before passing on to the detection heads. In particular, this module not only reduces the computational burden through lightweight convolutions but also adaptively highlights salient channels that are most critical for detecting small or partially occluded objects in aerial scenes.

**Fig. 4:**
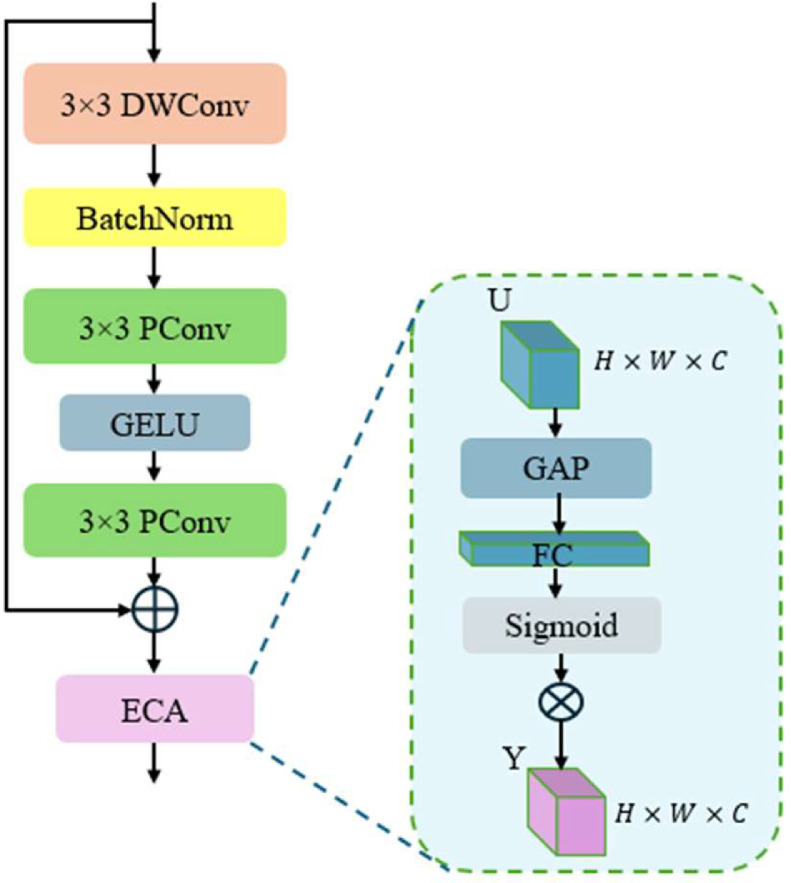
Internal structure of Attentive Inverted Bottleneck Aggregation (AIBA).

As shown in Fig. 4, the AIBA module begins with a 3 × 3 depthwise convolution (DWConv) for downsampling. This operation is followed by batch normalization for stable training and efficient scaling of feature responses. By employing a DWConv instead of a regular convolution, we preserve the overall spatial structure of the features while reducing computational overhead, especially beneficial in resource‐limited UAV edge platforms.

Next, we pass the intermediate output through an inverted bottleneck structure composed of two partial convolution (PConv) layers and a GELU nonlinearity in between. Let $X\in R^{H\times W\times C}$ denote the feature map entering this stage. The first Pconv compresses X into a lower‐dimensional representation, applies GELU activation, and then expands it back via the second Pconv:

(9) $$U=PConv_{2}\left(GELU\left(PConv_{1}\left(X\right)\right)\right)$$

Because partial convolution adaptively masks invalid or missing pixels in X, it ensures that blurred or occluded regions do not degrade the learned weights. This selective operation helps restore important details in UAV imagery and mitigates distortions caused by motion blur or partial occlusions.

Finally, an efficient channel attention (ECA) mechanism highlights discriminative channels in the refined output. We obtain a global descriptor $z\in R^{C}$ by applying global average pooling (GAP) along the spatial dimensions:

(10) z=GAP(U)

Then, z is fed into a lightweight 1D convolution of kernel size k to learn channel‐wise weights:

(11) $$w=\sigma \left(Conv1D_{k}\left(z\right)\right)$$

where σ(.) is the Sigmoid function. The final output Y is obtained via element‐wise multiplication of U with the attention weights w:

(12) Y=U⊗w

By focusing on the most critical channels for object detection, the ECA step further boosts the module’s representational power without substantially increasing parameter count.

In the neck portion of our real‐time UAV object detection framework, the AIBA module is replicated across multiple scales. Each scale receives features C3,C4, and C5 from the backbone and processes them through the AIBA for spatial-channel refinement. The resulting outputs serve as enriched multi‐level features, seamlessly integrated with other neck operations for top‐down and bottom‐up feature fusion. This design choice substantially improves detection performance on small or partially occluded objects, common in UAV scenarios, by leveraging the AIBA’s ability to emphasize relevant feature channels and selectively recover details. Moreover, the lightweight nature of the inverted bottleneck, partial convolutions, and efficient channel attention keeps computational overhead low, ensuring that the overall model can maintain real‐time inference rates on resource‐constrained hardware.

### Dual‐attention noise mitigation module

To bolster the reliability of our detector under the complex noise conditions encountered by UAVs, we propose a Dual‐Attention Noise Mitigation (DNM) module. Embedded within the multi‐scale neck, the DNM reduces extraneous interference and refines target‐relevant features at each scale. By performing complementary channel and spatial attention, this module substantially boosts detection robustness to environmental noise, such as glare, water reflections, or atmospheric perturbations, while maintaining a lightweight footprint suitable for real‐time inference on edge devices.

**Fig. 5:**
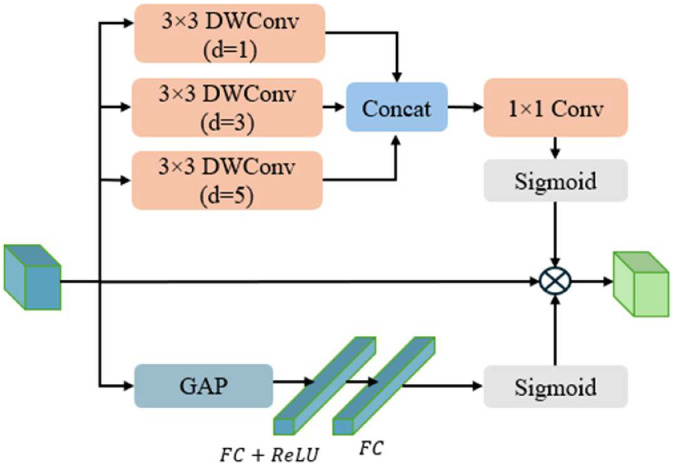
Dual-Attention Noise Mitigation (DNM) module, showing parallel channel and spatial attention pathways that collaboratively reduce environmental artifacts.

As illustrated in Fig. 5, the DNM applies two distinct yet synergistic attention mechanisms, channel attention and spatial attention, and then multiplies their results, along with the original input, to suppress noise and emphasize critical feature responses. In the channel attention branch, let $F\in R^{H\times W\times C}$ be the input feature map at a given scale. We begin by extracting a global representation $I\in R^{C}$ via global average pooling. A two‐layer fully connected (FC) network then transforms I to compute the per‐channel weights. Concretely,

(13) $$M_{C}\left(F\right)=\sigma \left(W_{1}\left(W_{0}\left(I\right)\right)\right)$$

where $W_{0}\in R^{\frac{C}{r}\times C}$ and $W_{1}\in R^{C\times \frac{C}{r}}$ are the learned parameters of the two FC layers, σ(.) is the Sigmoid activation, and r is the channel‐reduction ratio. This channel‐attention map $M_{C}\left(F\right)$ highlights channels that are most indicative of objects of interest, helping the network ignore channel‐wise noise artifacts.

In parallel, the same input F is convolved with multiple 3 × 3 depthwise convolutions, each employing a distinct dilation rate $d_{i}$. This strategy captures multi‐scale spatial context while minimizing the parameter count, because depthwise operations process each channel independently. The resulting feature maps are concatenated along the channel dimension and then fused by a 1 × 1 convolution. Finally, a sigmoid activation generates the spatial‐attention map $M_{S}\left(F\right)$:

(14) $$M_{S}\left(F\right)=\sigma \left(Conv_{1\;\times \;1}\left[DWConv_{3\;\times \;3,d_{i}}\left(F\right)\right]\right)$$

where $DWConv_{3\;\times \;3,d_{i}}\left(F\right)$ denotes a 3 × 3 depthwise convolution with dilation $d_{i}$. [.] indicates concatenation along the channel dimension, and σ(.) is the Sigmoid activation. $M_{S}\left(F\right)$ pinpoints key spatial locations in the feature map that contain objects or crucial cues, effectively suppressing spatially localized noise.

Finally, the DNM output is computed by combining these two attention maps with the original feature map:

(15) $$DNM\left(F\right)=M_{C}\left(F\right)⊗F⊗M_{S}\left(F\right)$$

where ⊗ denotes element‐wise multiplication.

Within our neck, each scale’s feature map undergoes the DNM module to eliminate noise before being fused with higher‐ or lower‐level features and used by prediction heads. This ensures that subsequent layers, such as feature concatenation blocks and detection heads, work with cleaner, more discriminative features. Crucially, the DNM adds minimal overhead; its channel‐reduction ratio r and lightweight spatial convolutions keep parameter growth and computation cost low. Consequently, it is well‐suited for real‐time UAV applications, where both computational resources and robust detection capabilities are highly valued.

## Method validation

### Evaluation protocol

To assess the performance of our proposed model comprehensively, we report per-class Average Precision (AP), the mean Average Precision (mAP), the model size in terms of parameters, computational complexity in Floating Point Operations (FLOPs), and inference speed in frames per second (FPS). Formally, AP is computed as the area under the Precision–Recall curve for each class c:

(16) $$AP_{c}=\int_{0}^{1}p\left(r\right)dr$$

where p(r) denotes the precision at recall r. The mAP is then obtained by averaging the AP values across all object categories:

(17) $$mAP=\frac{1}{C}\sum_{c=1}^{C}AP_{c}$$

where C is the total number of classes. Beyond accuracy measures, Parameters quantify the total learnable weights in the model, providing insights into memory footprint, while FLOPs gauge its computational complexity by counting the approximate number of floating-point operations needed per inference. Finally, FPS indicates real-time feasibility on a given hardware setup; higher FPS generally implies better suitability for time-sensitive UAV applications.

### Training protocol

All experiments were conducted on the RTX 4080 server with deployment tests on Jetson Orin Nano. Training utilized PyTorch with GPU acceleration. The AdamW optimizer was employed with an initial learning rate of 1 × 10^–4^, scheduled via cosine annealing, and a 5-epoch warmup. A batch size of 8 balanced throughput and GPU memory usage. Training proceeded for 100 epochs, with early stopping triggered by validation loss. Data augmentation strategies included random flips, rotations, cropping, and color jitter. Mixed precision (FP16) was enabled to improve efficiency.

### Comparison experiments with state-of-the-art detectors

In Table 2, we compare our proposed AAB-FusionNet against various state-of-the-art detectors on the DIOR dataset, which features 20 object categories captured in diverse aerial scenes. Our model achieves a mAP of 72.1 %, placing it on par with or outperforming other advanced methods such as YOLOv8 (70.7 %), RT-DETR (71.3 %), and FSoD-Net (71.8 %). Notably, AAB-FusionNet demonstrates particularly strong results on objects like ship (S), airport (AT), and windmill (WM), where maintaining discriminative cues over wide spatial contexts can be especially challenging. This gain is partly attributed to the AAB and the Multi-layer Feature Fusion Network, both of which foster better global and local context integration. Although Deformable-DETR obtains a slightly higher 72.2 % mAP, our approach nearly matches its performance while maintaining significantly lower computational costs. Such efficiency advantages are crucial for UAV-based applications, where resource constraints and real-time feedback loops demand careful balancing of accuracy and speed. In addition, our model’s results on smaller or partially occluded objects, such as bridge (B) or dam (D), highlight the utility of saliency-based feature extraction under cluttered aerial conditions. These improvements collectively indicate that AAB-FusionNet is well-suited for complex UAV environments, ensuring robust detection outcomes without sacrificing run-time efficiency.

**Table 2:** Detection performance on the DIOR dataset, comparing AAB-FusionNet with various state-of-the-art models in terms of mAP and per-class AP.

Method	mAP	AL	AT	BF	BC	B	C	D	ESA	ETS	GC	GTF	HB	O	S	SD	ST	TC	TS	V	WM
Faster R-CNN [3]	66.9	81.1	76.9	89.8	80.5	45.1	81.7	63.4	80.9	63.2	73.3	78.3	43.3	58.4	68.5	89.1	59	81.1	60.2	47.8	79.3
Cascade R-CNN [31]	67.4	81.2	81.4	90.1	81.1	46.9	81.5	68.1	84.2	65.3	74.5	81.6	37.8	60.4	68.9	88.8	60.5	81	57.5	47.7	80.7
RetinaNet [32]	66.1	53.3	77	69.3	85	44.1	73.2	62.4	78.6	62.8	67.2	76.6	49.9	59.6	71.1	68.4	45.8	81.3	55.2	44.4	85.5
YOLOv5 [9]	68.6	87.3	61.7	73.8	90	42.6	77.5	55.2	63.8	63.2	66.9	78	58.2	58.1	87.8	54.3	79.3	89.7	50.2	54	79.6
YOLOv8 [12]	70.7	87.6	74.3	76.3	87.3	40.7	77.6	60.3	68.2	60.4	76.3	80.3	55.2	53.4	83.4	58.4	70.2	85.3	53.4	50.6	78.6
FCOS [33]	61.2	73.5	68	69.9	85.1	34.7	73.6	49.3	52.1	47.6	67.2	68.7	46.3	51.1	72.2	59.8	64.6	81.2	42.7	42.2	74.8
CenterNet [34]	63.9	73.6	58	69.7	88.5	36.2	76.9	47.9	52.7	53.9	60.5	62.6	45.7	52.6	88.2	63.7	76.2	83.7	51.3	54.4	79.5
DETR [20]	68.6	39.6	74	65.2	80.7	26.5	75.2	66.8	70.5	52.5	74.2	62.1	27.4	47	8.5	46	14.5	64.4	54.3	14.4	55.6
RT-DETR [35]	71.3	88.1	75.6	77.2	88.7	41.3	78.4	62.2	70.3	61.4	78.2	83.3	57.6	55.1	85.8	60.3	73.1	87.6	55.2	53.1	80.4
DINO [36]	72.1	88.9	76.5	77.8	88.3	41.8	79.2	63.5	70.8	61.0	77.8	85.0	57.9	54.8	87.2	62.8	73.5	89.0	58.0	53.5	80.3
Deformable-DETR [21]	72.2	89.2	76.4	78.7	89.2	42.2	79.1	64.3	72.3	62.6	79.4	85.2	58.3	56.3	87.4	62.3	75.3	90.6	57.3	54.4	82.3
DIAG-TR [37]	72	68.4	72.2	71.9	85.1	29.8	76.8	60.2	70.3	73.2	75.1	35.5	35.5	50.8	41.6	71.8	32.9	81	53.1	33.8	78.6
ASSD [38]	71.1	85.6	82.4	75.8	89.5	40.7	77.6	64.7	67.1	61.7	80.8	78.6	62	58	84.9	76.7	65.3	87.9	62.4	44.5	76.3
FSoD-Net [39]	71.8	88.9	66.6	86.6	90.2	45.5	79.6	48.2	86.9	75.5	67	77.3	53.6	59.7	78.3	59.9	77.3	78.6	48.2	52	90.6
Ours	72.1	89.3	76.6	79.2	88.9	45.3	79.4	59.1	68.4	63.1	80.2	89.7	60.3	57.2	87.2	61.1	73.3	89.8	58.6	55.1	81.8

Table 3 presents results on the RSOD dataset. AAB-FusionNet attains a mAP of 93.0 %, thereby outperforming or closely matching the top contenders such as DINO (92.5 %) and RT-DETR (91.1 %). This dataset, focused on four primary categories (aircraft, oil tank, overpass, and playground), demands a model that can adapt to variable object sizes and shapes often observed in aerial images. Our method’s detailed design proves effective for retaining spatial details when objects vary significantly in scale or exhibit unique shapes. The breakdown of per-class AP reveals especially high precision in detecting aircraft (94.7 %) and oil tanks (97.4 %), two categories that can appear in clustered formations and require advanced modeling of local structures. Although our result for overpass (80.8 %) is slightly lower than on some other categories, it remains competitive with state-of-the-art models that typically struggle with complex backgrounds and partial occlusions. These findings reinforce the notion that AAB-FusionNet effectively balances multi-scale feature representations and saliency-driven attention to handle the myriad challenges found in remote sensing imagery, ultimately leading to consistently superior or comparable detection outcomes across diverse aerial datasets.

**Table 3:** Benchmark results on the RSOD dataset, contrasting AAB-FusionNet’s detection accuracy with leading approaches across four core object categories.

Method	mAP	Aircraft	Oil tank	Overpass	Playground
Faster R-CNN [3]	84.47	70.84	90.19	78.74	98.09
Cascade R-CNN [31]	91.3	94.2	96.1	83.2	99
YOLOv5 [9]	89.5	88.9	95.2	80.3	98.7
YOLOv8 [12]	90.1	90.5	95.7	81.5	98.9
FCOS [33]	86.2	76.3	92.8	79	98.2
DETR [20]	87.8	75.5	93.5	79.8	98.5
RT-DETR [35]	91.1	93.2	96	82.9	99
DINO [36]	92.5	94	96.5	84.5	99.3
Deformable-DETR [21]	90.7	92.5	95.8	83.5	99.2
Ours	93.0	94.7	97.4	80.8	99.1

Table 4 provides computational comparisons that illustrate how our method stands out in terms of efficiency and speed. Despite delivering top-tier accuracy, AAB-FusionNet maintains a parameter count of 28.6 M and FLOPs of 48.3 G, which are substantially lower than some other high-performing models, such as Cascade R-CNN (63.5 M parameters, 140.2 G FLOPs) and DINO (47 M parameters, 210 G FLOPs). This streamlined design directly results in a significantly higher inference speed of 45 FPS, outperforming most competing detectors in the table. Crucially, the improved throughput does not come at the expense of detection quality, underscoring the success of our saliency-based attention and efficient feature fusion modules. By striking a favorable balance between accuracy and computational load, AAB-FusionNet demonstrates its strong potential for real-time UAV edge computing platforms, where both resource constraints and dependable detection performance are paramount.

**Table 4:** Comparison of computational costs and speed among top-performing detectors, detailing parameters, FLOPs, and inference FPS.

Method	Parameters (M)	FLOPs (G)	FPS
Faster R-CNN [3]	41.17	69.29	22
Cascade R-CNN [31]	63.5	140.2	14
RetinaNet [32]	37.7	97.8	18
YOLOv5 [9]	46.5	116	28
YOLOv8 [12]	43.7	118.9	30
FCOS [33]	32	133.3	20
DETR [20]	41	187	11
RT-DETR [35]	41	136	21
DINO [36]	47	210	9
Deformable-DETR [21]	40.5	173	13
Ours	28.6	48.3	45

### Visualization of detection results

**Fig. 6:**
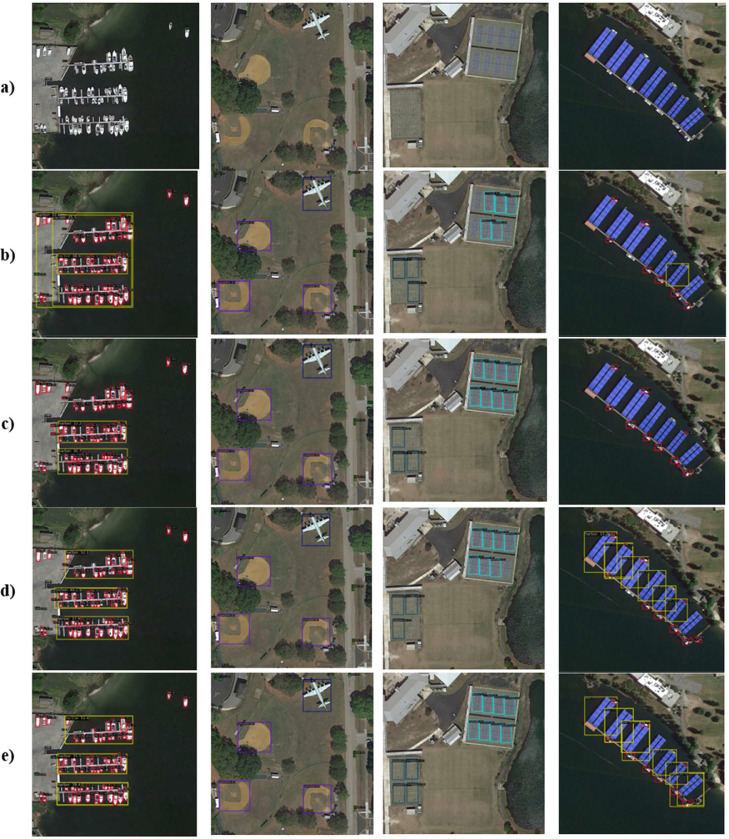
Visualization of detection outcomes on sample aerial images; (a) the original image, (b) Faster R-CNN, (c) YOLOv8, (d) Deformable-DETR, and (e) our AAB-FusionNet. Notice how AAB-FusionNet consistently captures small or partially occluded objects with high precision.

Fig. 6 illustrates side-by-side detection outcomes for multiple methods, specifically, (a) original aerial images, (b) Faster R-CNN, (c) YOLOv8, (d) Deformable-DETR, and (e) our proposed AAB-FusionNet. A quick glance reveals that Faster R-CNN sometimes overlooks smaller objects near the edges of each scene, leading to incomplete coverage. YOLOv8 captures more instances of these small targets but occasionally misaligns bounding boxes in areas with complex backgrounds, such as marinas. Deformable-DETR performs well across large and medium-sized objects, yet it occasionally produces false positives or slightly irregular bounding boxes in cluttered regions. By contrast, AAB-FusionNet maintains consistently precise detections across all object scales; as seen in subfigure (e), bounding boxes closely match the outlines of targets, even when they are partially occluded or adjacent to similar structures. In the second and third columns, which contain aircraft and other mid-scale features, AAB-FusionNet exhibits robust delineation of object boundaries with minimal overlaps or missed regions. This fidelity extends to the final column, where floating objects are arranged in tight clusters, our method succeeds in isolating each unit without merging multiple objects into a single detection. Overall, these visual comparisons suggest that AAB-FusionNet successfully balances large-scale context awareness with fine-grained, saliency-driven focus, allowing it to excel in complex aerial scenes.

**Fig. 7:**
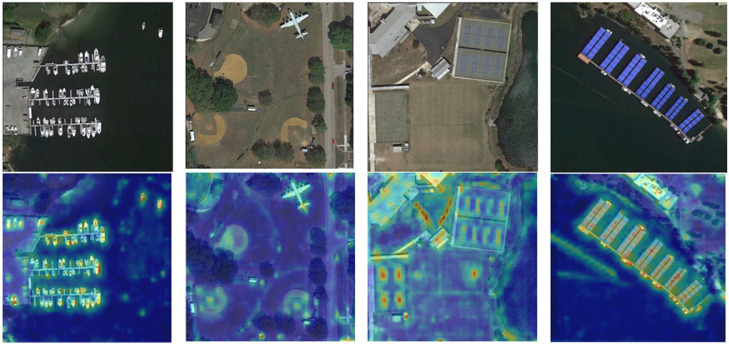
Heatmaps generated by our model, highlighting regions deemed most salient for detection.

Fig. 7 presents heatmaps generated by our model, demonstrating how the model localizes crucial regions for object recognition. High-intensity areas in the heatmap align with key structural cues, such as the outline of boats, airplane wings, or solar panel grids, indicating that the model is effectively directing its computational resources toward features most indicative of the target class. Notice that even in cluttered environments, like the marina or the busy sports field, the saliency distributions remain sharply concentrated around relevant objects, implying minimal distraction from the background. Meanwhile, the presence of moderate to low intensities over relatively uniform or irrelevant regions confirms that ASA effectively downplays background noise. By comparing these heatmaps to the bounding box predictions in Fig. 6, one can see a close correspondence between high-saliency zones and correct object detections. This alignment underscores how our saliency-aware design ensures more robust, accurate results under the challenging conditions typical of UAV-based aerial imagery.

### Ablation study

To evaluate the impact of each component in AAB-FusionNet, we conduct a series of ablation experiments on the RSOD dataset. We gradually incorporate key modules into a baseline network that retains only the essential MBConv blocks in its backbone. As shown in Table 5, we measure the mAP as well as model complexity in terms of Parameters, FLOPs, and FPS. These experiments clarify how each proposed module contributes to overall performance while balancing computational overhead.

**Table 5:** Ablation study on the RSOD dataset. Each row incrementally integrates a proposed module, showing the effect on mAP, parameter count, FLOPs, and FPS.

Configuration	ASA	AIBA	DySample	DNM	mAP ( %)	Parameters (M)	FLOPs (G)	FPS
Baseline (MBConv Only)	–	–	–	–	90.1	24.0	37.0	50
+ ASA	✓	–	–	–	90.8	26.0	39.5	47
+ ASA + AIBA	✓	✓	–	–	91.4	26.5	41.1	46
+ ASA + AIBA + DySample	✓	✓	✓	–	92.0	27.1	44.9	46
+ ASA + AIBA + DySample + DNM	✓	✓	✓	✓	93.0	28.6	48.3	45

Baseline (MBConv Only): Removing ASA from the backbone and omitting all specialized fusion or noise mitigation modules yields a network capable of 90.1 % mAP, requiring 24.0 M parameters and 37.0 G FLOPs, with a throughput of 50 FPS. Although this lightweight version is fast, it struggles to capture nuanced details in cluttered aerial scenes.

+ ASA: Integrating the ASA mechanism in the backbone elevates mAP to 90.8 %, at the cost of a slight increase in parameters (26.0 M) and FLOPs (39.5 G), reducing inference speed to 47 FPS. The performance gain underscores ASA’s ability to highlight discriminative features, particularly for challenging object instances in aerial imagery.

+ ASA + AIBA: Adding the AIBA module further refines multi-scale features, raising mAP to 91.4 % with a modest increase in complexity (26.5 M parameters, 41.1 G FLOPs) and a small decrease to 46 FPS. These results suggest that AIBA effectively recovers spatial details and channels pertinent to small or occluded objects.

+ ASA + AIBA + DySample: By incorporating DySample for adaptive feature alignment, the model reaches 92.0 % mAP, with parameters now at 27.1 M and FLOPs at 44.9 G. Although inference speed remains at 46 FPS, improved feature consistency across scales facilitates better localization and classification, particularly when target sizes vary considerably.

+ ASA + AIBA + DySample + DNM: Finally, enabling the DNM module boosts performance to 93.0 % mAP, while the parameter count and FLOPs increase to 28.6 M and 48.3 G, respectively. Inference speed remains competitive at 45 FPS. The synergy of channel and spatial attention mechanisms in DNM effectively filters environmental artifacts, further improving detection robustness.

Overall, these ablation experiments verify that each module, ASA, AIBA, DySample, and DNM, makes a quantifiable contribution to accuracy while introducing manageable computational overhead. Notably, the final configuration delivers a favorable balance between performance and efficiency, reinforcing AAB-FusionNet’s suitability for real-time UAV edge computing applications.

### Embedded platform evaluation

To evaluate the feasibility of AAB-FusionNet in constrained environments typical of UAV edge computing, we deployed it on the NVIDIA Jetson Orin Nano and assessed its performance on the RSOD test set. We measured four key metrics: mAP, FPS, Power (W), and Efficiency (FPS / W). All competing methods were optimized using TensorRT-based FP16 precision on the Orin Nano to ensure a fair comparison. All images from the RSOD validation subset were processed, and we report average results across three independent runs.

As shown in Table 6, YOLOv5 achieves an mAP of 89.5 %, running at 13 FPS with a power consumption of 12.2 W, leading to an efficiency of 1.1 FPS/W. YOLOv8 improves precision to 90.1 % at 25 FPS, drawing 13.4 W and attaining 1.9 FPS/W. Our proposed AAB-FusionNet surpasses both methods in terms of detection accuracy, achieving an mAP of 93.0 %, while maintaining an inference speed of 30 FPS at 14.6 W, resulting in 2.1 FPS/W. Although it does not match the raw throughput of higher-FPS solutions on the Orin Nano, AAB-FusionNet’s notable gain in mAP underscores its reliability for precise detection in aerial scenarios.

**Table 6:** Embedded platform evaluation on the NVIDIA Jetson Orin Nano, highlighting mAP, inference speed (FPS), power consumption (W), and efficiency (FPS/W) for each method tested on the RSOD dataset.

Method	mAP ( %)	FPS	Power (W)	Efficiency (FPS / W)
YOLOv5 [9]	89.5	13	12.2	1.1
YOLOv8 [12]	90.1	25	13.4	1.9
AAB-FusionNet (Ours)	93.0	30	14.6	2.1

Overall, the deployment results on the Jetson Orin Nano confirm that AAB-FusionNet meets the stringent demands of UAV edge computing, where power efficiency, real-time responsiveness, and robust detection accuracy must coexist. By combining carefully tuned attention mechanisms with multi-scale feature fusion, our model demonstrates both high precision and rapid inference. These findings highlight its potential for real-world aerial applications, such as infrastructure inspection and disaster management, in which on-board processing speed and energy considerations are paramount.

## Conclusions and future work

In this paper, we introduced AAB-FusionNet, a real-time object detection framework tailored for UAV edge computing platforms. By incorporating the Adaptive Attention Block (AAB), which combines the Adaptive Saliency-based Attention (ASA) module with MBConv sub-layers, the proposed network can selectively emphasize critical regions in aerial imagery while preserving computational efficiency. Additionally, our Multi-layer Feature Fusion Network leverages Attentive Inverted Bottleneck Aggregation (AIBA), DySample, and the Dual-Attention Noise Mitigation (DNM) module to effectively integrate multi-scale features and suppress noise. Experimental results on multiple aerial datasets demonstrated that AAB-FusionNet consistently achieves robust detection performance, particularly for challenging scenarios involving occlusions and small objects, without compromising real-time inference speeds on resource-constrained UAV hardware. Future work will focus on several avenues to further enhance the model’s capabilities and address emerging challenges in UAV-based perception systems. First, integrating domain adaptation strategies could improve performance when operating across diverse environmental and weather conditions. Second, more advanced hardware-aware optimization techniques, such as quantization, mixed-precision training, or model pruning, may reduce the computational footprint and latency even further, broadening the scope of real-time applications. Third, extending the method to related tasks such as object tracking, semantic segmentation, or 3D localization could further enrich the autonomy and situational awareness of UAV platforms. By exploring these directions, we aim to refine AAB-FusionNet into a more versatile, efficient, and robust solution for real-time aerial perception.

## Limitations

Despite achieving strong detection results on benchmark datasets, the proposed AAB-FusionNet has not been extensively validated under varying real-world flight conditions where factors like extreme weather, abrupt motion blur, and sudden lighting changes can significantly affect performance. Additionally, the framework’s reliance on a specific set of UAV hardware configurations may limit its immediate applicability to different edge devices without careful re-optimization or retraining.

A further limitation lies in the specialized modules (e.g., ASA, AIBA, and DNM), which increase model complexity and may pose challenges for seamless integration into existing pipelines. The current approach also assumes access to well-annotated aerial datasets, potentially constraining its efficacy in domains or scenarios with scarce labeled data.

## Ethics statements

This study did not involve human participants, animal subjects, or data obtained from social media platforms. The aerial imagery used for model development and testing was sourced entirely from publicly available datasets. Accordingly, no personal data were collected, and no additional ethical approvals were necessary for this research.

## Supplementary material *and/or* additional information [OPTIONAL]

None

## Related research article

*None*

## For a published article

*None*

## CRediT authorship contribution statement

**Chi Kien Ha:** Conceptualization, Methodology, Data curation, Validation, Formal analysis. **Hoanh Nguyen:** Conceptualization, Methodology, Data curation, Validation, Formal analysis. **Long Ho Le:** Methodology, Software, Formal analysis, Validation, Visualization, Writing – original draft.

## Declaration of competing interest

The authors declare that they have no known competing financial interests or personal relationships that could have appeared to influence the work reported in this paper.

## Data Availability

Data will be made available on request.
